# Slow Diaphragmatic Breathing for Chronic Migraine Prevention and Treatment: A Case Report

**DOI:** 10.3390/reports9020140

**Published:** 2026-04-29

**Authors:** Tanya G. K. Bentley, Gina M. D’Andrea-Penna, Emily L. Hightower

**Affiliations:** 1 Health and Human Performance Foundation, 1223 Wilshire Blvd, Suite 570, Santa Monica, CA 90403, USA; 2 UC San Diego Neurosciences Graduate Program, 9500 Gilman Drive, La Jolla, CA 92093, USA

**Keywords:** case report, migraine, slow diaphragmatic breathing, breathing practices, autonomic dysfunction, migraine prevention

## Abstract

**Background and Clinical Significance:** Migraine is a common yet debilitating condition that significantly impacts personal lives, productivity, and the healthcare system. Pharmacological interventions provide relief for some migraine sufferers, but for others, are ineffective or accompanied by side effects. Emerging evidence implicates autonomic nervous system dysfunction in migraine pathophysiology, suggesting that mind–body interventions may offer a simple, cost-free therapeutic option. **Case Presentation:** A 61-year-old woman presented with severe daily migraines that had persisted for years despite medication and dietary changes. Upon starting a regular 10 min slow diaphragmatic breathing practice, her migraines ceased immediately. At a 12-month follow-up, she had only experienced two minor headaches and reported improvements in both daily functioning and quality of life. **Conclusions:** These findings underscore the potential role of autonomic imbalance in chronic migraine and the preliminary feasibility of breathing interventions as an accessible, low-risk treatment that may, for some, surpass medication in efficacy. Breathing practices may offer a viable alternative to pharmaceutical interventions that benefits both patients and healthcare systems alike.

## 1. Introduction and Clinical Significance

Migraine is the third-most-common illness in the world, afflicting over one billion people globally [1]. This neurological disease can cause severe pain, nausea, vomiting, disorientation, and sensory hypersensitivity. Linked to both stress and estrogen, migraine affects females two to three times more frequently than males [1]. Pathophysiology of migraine is not fully understood but involves dysregulation across multiple interacting systems, such as trigeminovascular pathway activation, central sensitization, hypothalamic hyperactivity, neuroinflammation, and autonomic imbalance [2,3,4]. A wide variety of treatments for migraine exist. Amongst the most popular pharmacological options are nonsteroidal anti-inflammatory drugs (NSAIDs), triptans, β blockers, tricyclic antidepressants, anticonvulsants, Botox, and calcitonin gene-related peptide (CGRP) inhibitors [3,5,6]. Yet, despite these numerous prevention and treatment medication options, migraines remain a significant public health issue and a leading cause of disability [7,8].

Stress, sleep disturbances, alcohol, and specific foods are the most commonly reported migraine triggers [9], highlighting the potential role of lifestyle in migraine prevention. Non-pharmacological interventions such as anti-inflammatory, Mediterranean, and ketogenic diets [10], regular aerobic exercise [11], and stress management via relaxation training, biofeedback, and cognitive-behavioral therapy [12] have shown promise for migraine prevention. Beyond removing migraine triggers, these interventions may collectively reduce physiological strain from chronic psychological and physical stressors [13]. Continued or repeated exposure to stress may play a key role in migraine chronification due to resulting maladaptations in central and peripheral physiology that further dysregulate the stress response, pain processing, and neuronal excitability [14].

Conscious breath control directly affects the nervous system. Slow diaphragmatic breathing with extended exhales and brief breath-holds may help attenuate heightened sympathetic nervous system activity and improve autonomic flexibility in individuals with migraines [15,16]. While various studies have examined complementary and integrative medicine approaches for migraines such as yoga, meditation, and acupuncture [17], few have investigated the therapeutic potential of breathing practices [18,19]. This case report, prepared according to the CARE guidelines [20] (Table A1) and with informed consent, aims to assess the feasibility of a self-paced slow breathing intervention for chronic migraine and its potential efficacy in reducing migraine frequency, duration, and intensity.

## 2. Case Presentation

### 2.1. Case Description

The patient, an Italian-American female aged 61 years at the time of intervention, had experienced migraines for 34 years and reported a maternal history of menopause-related migraines. She reported being a non-smoker and consuming two alcoholic drinks, four nights per week. As a remote executive assistant, she sat at a computer from 8 AM to 5 PM every weekday, with minimal short breaks. She walked, jogged, and lifted weights three days per week and meditated twice per month. Before learning the breath intervention described in this report, the patient had not tried breathing practices.

**Figure 1:**
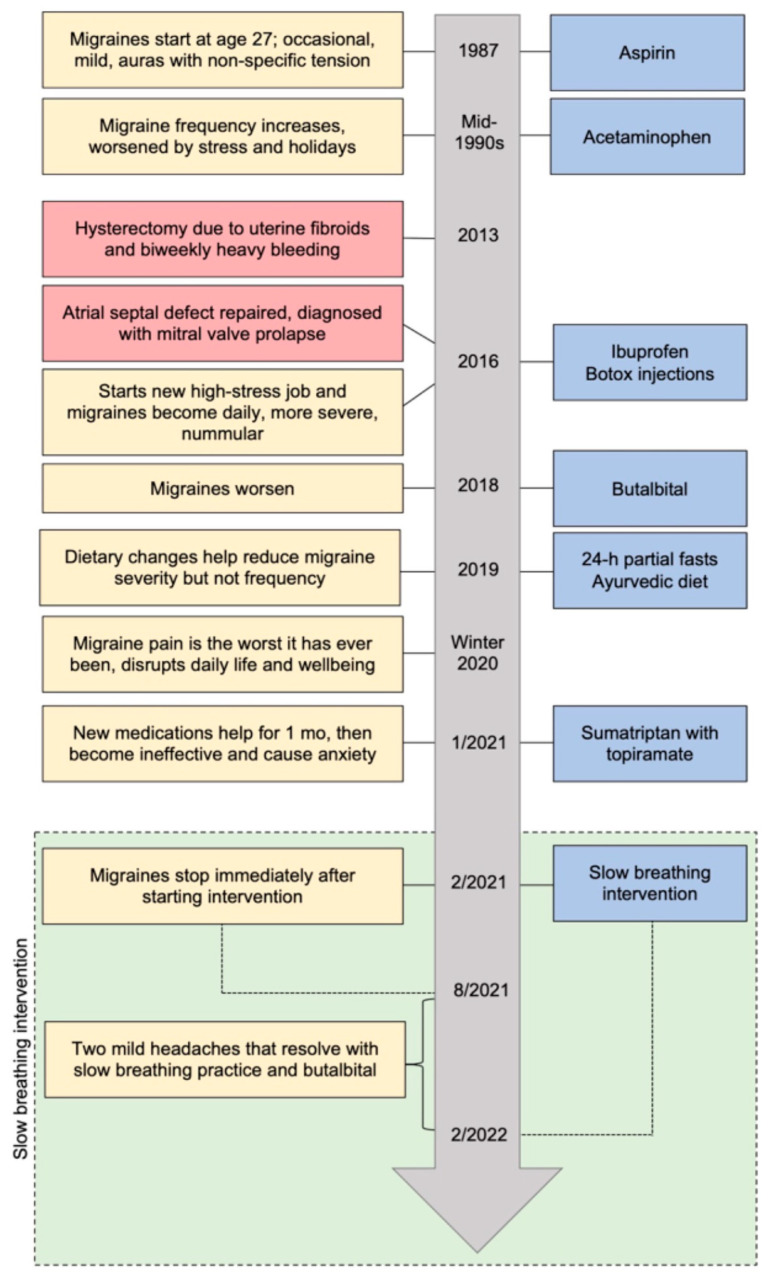
Patient timeline. Yellow boxes include migraine symptoms, red boxes include other medical events, blue boxes include migraine treatments, and the green box represents the intervention period. Migraine treatments in blue boxes are placed at the time period in which the patient started each medication, but intermittent use of acetaminophen, aspirin, ibuprofen, and butalbital occurred throughout the period preceding the slow breathing intervention. h, hour; mo, month.

At age 27, the patient began having occasional stress-related migraines with auras and non-specific tension that were manageable with baby aspirin (Figure 1). When they became more consistent in her mid-30s, her neurologist diagnosed her with migraine based on her symptoms and medical history. A standard dosage of acetaminophen was effective when aspirin at onset was inadequate. Stress and the holidays exacerbated symptoms, although they were never debilitating nor caused her to miss work or social events. A hysterectomy in 2013 to address uterine fibroids and biweekly heavy bleeding did not affect migraine frequency or severity. In 2016, the patient had a newly diagnosed atrial septal defect repaired, was diagnosed with mitral valve prolapse, and started a new position at work.

Upon starting this new, high-stress job, the patient’s migraines became daily and more severe, involving recurrent pain on the top left side of her head without aura. At this point, the patient underwent an MRI, which showed scarring consistent with migraines but no other findings. Thereafter, her neurologist diagnosed her with chronic migraine as well as nummular headache based on the localization of pain. Onset was consistently during acute work stress and still worsened during the winter holidays.

These migraines generally lasted all day and were rated by the patient mostly as a 5, or occasionally a 9 or 10, on a scale of 1–10. Although the patient identified work stress as the main trigger, she rarely missed work for fear of exacerbating stress. She typically stayed through the workday in debilitating pain and returned home incapacitated. The migraines disrupted time with friends and family, holiday travel, and hobbies. Severity worsened over a five-year period.

During this time, taking 600 mg of ibuprofen at migraine onset was the patient’s first-line treatment, typically enabling three hours of focused work with somewhat reduced pain, though its ongoing use gradually led to intolerable gastrointestinal discomfort. Standard height-and-weight-adjusted dosages of prescriptive medicines, including those in the sumatriptan family, and three rounds of botox injections offered no relief. During severe episodes, she paired butalbital, taken in standard dosage, with ibuprofen, which she reported worked the best of all attempted pharmacological treatments. Sumatriptan with topiramate, which she tried in standard dosages in January 2021, worked for one month and subsequently caused anxiety; she stopped using it after two months. She continued intermittent use of aspirin, acetaminophen, ibuprofen, and butalbital throughout the period preceding the breathing intervention despite their limited effectiveness.

In addition to pharmaceutical interventions, in February 2019, the patient began practicing weekly 24 h partial fasts of bone broth by day and a smoothie for dinner. She also incorporated an ayurvedic approach to her diet. These changes reduced migraine severity but not frequency. By the winter holiday season of 2020, the migraine pain had intensified further, preventing her from seeing friends and family and from living her normal life. Frustrated with the intractable pain and brain fog, she described her daily migraines as a “recurring nightmare.” The patient became isolated and depressed. She missed weekend activities to stay in bed and recover, albeit knowing that the migraines would return during work-week stress.

### 2.2. Treatment Pathway

**Figure 2:**
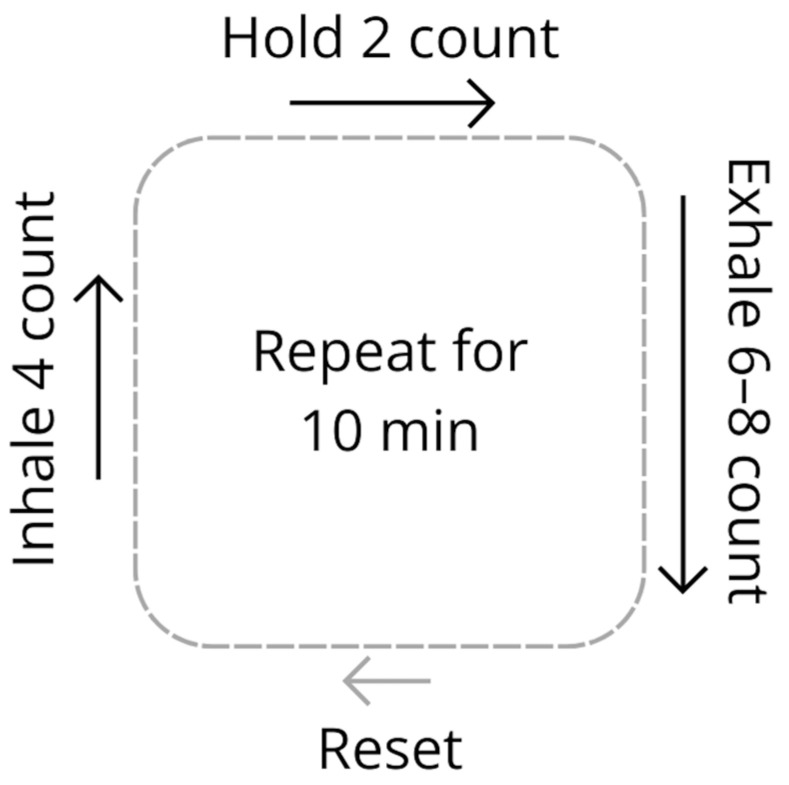
Slow diaphragmatic breathing intervention. The patient was prescribed a practice of slow diaphragmatic breathing with extended exhales and brief breath-holds, in which she was instructed to inhale for a comfortable count of four, pause for a count of two, exhale for a count of six or eight, and take a brief, natural pause to reset between breath cycles before proceeding with the next inhale. The instruction encouraged the patient to self-pace by counting faster or slower based on her body’s signals while maintaining the prescribed inhale-pause-exhale ratio. She was instructed to continue this pattern for 10 min. This practice was to be used both daily as a prophylactic and upon stress or headache onset. min, minutes.

In February 2021, a practitioner trained in nervous system rehabilitation and therapeutic breathwork provided the patient with a slow diaphragmatic breathing practice to prevent and treat migraines. Prior to working with the patient, the practitioner had observed positive outcomes from breath practice implementation in other migraine sufferers but had not methodically evaluated these effects. Since stress was the patient’s primary trigger, the practice was designed to tame the autonomic nervous system’s stress response via slow, extended-exhale breathing with brief breath-holds. For 10 min, the patient was instructed to inhale for a comfortable count of four, pause for a count of two, exhale for a count of six to eight, and reset between breath cycles with a natural pause (Figure 2). The instruction introduced extended exhales before adding breath holds and encouraged self-pacing through counting faster or slower based on her body’s signals while maintaining the prescribed inhale-pause-exhale-reset ratio. If the patient felt strain during the practice, she could count faster; if she felt rushed or had extra air and time, she could count slower. She was guided to use optimal respiratory diaphragm mechanics, allowing her lower ribs to gently lift and expand on inhales and gradually descend on exhales. The practice was performed breathing through the nose.

Downloadable MP3 guidance (Appendix A.1) and written instructions (Appendix A.2) were delivered via email and discussed via phone call. The patient was instructed to complete the practice in a comfortable seated or lying-down position in a quiet, distraction-free, darkened room at the first sign of, or during, a migraine. She was also instructed to do it prophylactically either every morning upon waking or every evening before bed, as well as when feeling stressed or anxious. The practice was sufficiently simple that the patient, soon after starting a daily practice, could implement it as needed without audio guidance. From the start, she felt comfortable using a cadence with a four-count inhale, two-count pause, and eight-count exhale with a reset, which she followed throughout the intervention.

The practitioner followed up with the patient via email at two weeks and at 2, 6, and 12 months to assess compliance, answer questions, and collect data regarding migraine frequency, severity, duration, and impact on daily living.

### 2.3. Outcomes

Upon receiving the audio recording and instructions, the patient performed the breathing practice daily and whenever she felt stress or headache onset. For the first week, she used the recording before leaving bed each morning. From week two onward, she felt comfortable applying the practice without the recording. At this point, she also began using it throughout the day upon experiencing stress or migraine-onset symptoms. Over the next two months, she practiced several times per week to reduce stress and manage potential migraine onset. She reported using it every time she noticed headache symptoms. The practice became simple and easy for her to do without the recorded instructions and in any setting, whether at work, in her car, at home, or elsewhere. At 12 months and to the time of this reporting, the patient has integrated the breathing practice into her lifestyle, performing it once or twice a week upon stress or headache symptoms or as a morning meditative practice (Table 1).

The patient’s migraines ceased completely and immediately after the first day of starting the breathing practice. Since then, the patient has not had a single debilitating migraine. During the 12-month follow-up, she reported two mild migraines that came as a surprise and for which she could specify no trigger beyond normal work stress. Both migraines were fully relieved with butalbital combined with the breathing practice.

Since starting the breath practice, the patient has reported feeling more in control of headache symptoms, and migraines no longer impact her activities of daily living. Despite continued work stress, she reported functioning at a higher level, unencumbered by migraine pain. She reported a significant quality of life improvement, finally able to travel and see family over the holidays. No adverse effects occurred during or after the breathing practice for the duration of follow-up. She keeps butalbital on hand as a “safety net” but can avoid migraines by using the breathing practice at the first sign of onset.

## 3. Discussion

This case study demonstrates the potential value of slow diaphragmatic breathing as a simple, accessible, no-cost approach to migraine treatment and prevention. The patient suffered a decades-long burden of migraines, which became daily and debilitating in the five years preceding the intervention described here. A regular 10 min breathing practice with brief breath holds and extended exhales provided immediate relief and virtually eliminated her migraines, with only two mild ones reported at 12-month follow-up.

This breathing practice was always available to the patient and required no prescription or equipment, allowing her to take agency over her stress and symptoms in real time. The intervention taught the patient to notice her body’s signals and regulate her breathing, empowering her to tame her previously intractable stress response and, consequently, to eliminate not only migraines but also headache-anticipation anxiety.

Psychological stress has the potential to affect a variety of pathways and processes implicated in migraine pathogenesis. Chronic stress not only disrupts hypothalamic and autonomic function but also can lead to inflammation, hyperalgesia, trigeminal pathway activation, and central sensitization [21,22]. Some have posited that chronic migraine is, in fact, a disorder of allostatic load, in which chronic and cumulative stressors induce progressive maladaptations across the brain–body system [14,23]. The current case aligns with these allostatic accounts and with the potential of mind–body interventions to restore such systemic “balance [17]”.

While prior studies examine therapeutic effects for migraine of yoga, tai chi, and meditation [9], which often integrate breath practices, few have evaluated regulated breathing in isolation. Of those that have, all have employed more technically complex breathing practices, including alternate-nostril breathing [18] and pranayama [19], and involved more extensive training. A separate body of evidence finds stress-reducing effects of diverse forms of slow breathing in populations with high anxiety and clinical conditions, from chemotherapy to cardiac surgery [24]. This case report is one of the first to merge both lines of research, unveiling the benefits of regulated breathing for migraines as well as stress.

Interestingly, the breathing intervention in this report surpassed prescription medications in efficacy. For some, medications provide inadequate relief and engender unpleasant side effects. Moreover, symptomatic overuse of some migraine medications can contribute to chronic daily headaches [25]. Turning to breathing as a first-line treatment may help reduce dependency on medications and increase patient self-efficacy, improving migraine symptoms and quality of life. Unlike pharmaceutical interventions, slow breathing is free of cost and side effects, offering a safer, more affordable alternative.

It is notable that the practice described here emphasized pace flexibility, in which the patient determined a comfortable breathing cadence based on her bodily signals and individual needs. Such self-pacing is likely more beneficial than following a predetermined pace, which may elicit air hunger, tension, and over-breathing, inadvertently exacerbating stress and symptoms.

As with any case study, this report demonstrates intervention success in this patient only. More research is needed to evaluate effectiveness in other migraine sufferers. The patient described here was both able and willing to perform the prescribed practice with minimal guidance, integrating it into her lifestyle after one week of audio-guided training. More extensive, in-person guidance may be necessary for those with breath sensitivities or who have difficulty engaging their diaphragm due to poor breathing mechanics or clinical complications.

While the simplicity and brevity of breathing practices lower the barrier to compliance, individuals with shorter, milder migraine histories, less frustration with failed treatments, or reduced levels of motivation or energy may be less adherent. Lack of consistent, ongoing practice may not yield lasting improvement in migraine symptoms and stress.

Psychological stress commonly contributes to migraines and was the patient’s primary, if not only, trigger. Nonetheless, some migraineurs have external triggers, like food, exercise or sleep changes; and lifestyle factors such as these can influence cumulative stress burden [13] and migraines [16]. Individuals with health risk behaviors or triggers other than emotional stress may experience limited benefits from this practice in absence of larger behavioral changes.

Lastly, the current study does not allow one to disentangle the effects of the described breathing practice from the placebo effect or impact of moving to a quiet, darkened environment. However, given the patient’s prolonged history of ineffective treatments, a placebo effect is likely to be minimal, as prior research observes lower placebo response rates in migraine patients who have experienced failed treatments in the past [26,27,28]. Similarly, voluntary breath control exerts unique neurophysiological benefits that cannot be replicated by a low-stimulation environment alone. Slow, deep breathing influences autonomic balance and brain state both in a bottom-up fashion–via pulmonary stretch receptors [29,30], respiratory sinus arrhythmia [31,32], and gas exchange [33]–and through a global respiratory rhythm that synchronizes neuronal populations and shapes network dynamics [34]. The magnitude of response observed in this case thus appears difficult to attribute solely to a placebo effect or change in environment, though future research that includes blinding and biochemical analyses can better investigate these nuances and clarify the neurophysiological underpinnings of intervention efficacy.

## 4. Conclusions

This case report illustrates the feasibility and efficacy of a slow breathing practice in a patient with chronic debilitating migraines for whom medications had failed. In doing so, it reinforces the role of autonomic dysregulation in migraine pathophysiology and points toward voluntary breath control as a means of mitigating stress-induced neural maladaptations. Given the simplicity and accessibility of such practices, more research is needed to assess the therapeutic potential of regulated breathing for migraine prevention and treatment. Should these findings generalize to other migraine sufferers, breath interventions may offer a viable alternative to medication that provides immediate, long-term relief. This safe, cost-free treatment may offer broad-ranging benefits to patients and healthcare systems alike.

## Figures and Tables

**Table 1:** Patient’s breath practice implementation and migraine outcomes over 12-month follow-up. w/, with; w/o, without; wk, week.

Intervention Stage	Breath Practice Implementation	Migraine Symptoms	Adverse Effects
Week 1	Every morning w/audio guidance	None	None
Weeks 2–4	Every morning w/o audio; during day upon stress/headache onset	None	None
Months 2–6	Several times weekly to reduce stress or manage potential migraine onset	None	None
Months 6–12	1–2x/wk upon stress or potential migraine onset; as morning practice	2 mild headaches that subsided w/breath practice + butalbital	None

## Data Availability

Data are contained within the article.

## Appendix A

**Table A1:** CARE Checklist [20].

Topic	Item	Checklist Item Description	Included?
Title	1	The diagnosis or intervention of primary focus followed by the words “case report”	yes
Key words	2	2 to 5 key words that identify diagnoses or interventions in this case report, including “case report”	yes
Abstract (no references)	3a	Introduction: What is unique about this case and what does it add to the scientific literature?	yes
	3b	Main symptoms and/or important clinical findings	yes
	3c	The main diagnoses, therapeutic interventions, and outcomes	yes
	3d	Conclusion—What is the main “take-away” lesson(s) from this case?	yes
Introduction	4	One or two paragraphs summarizing why this case is unique (may include references)	yes
Patient information	5a	De-identified patient specific information	yes
	5b	Primary concerns and symptoms of the patient	yes
	5c	Medical, family, and psycho-social history including relevant genetic information	yes
	5d	Relevant past interventions with outcomes	yes
Clinical findings	6	Describe significant physical examination (PE) and important clinical findings	yes, based on patient report
Timeline	7	Historical and current information from this episode of care organized as a timeline	yes
Diagnostic assessment	8a	Diagnostic testing (such as PE, laboratory testing, imaging, surveys)	not applicable
	8b	Diagnostic challenges (such as access to testing, financial, or cultural).	not applicable
	8c	Diagnosis (including other diagnoses considered)	not applicable
	8d	Prognosis (such as staging in oncology) where applicable	not applicable
Therapeutic intervention	9a	Types of therapeutic intervention (such as pharmacologic, surgical, preventive, self-care)	yes
	9b	Administration of therapeutic intervention (such as dosage, strength, duration)	yes
	9c	Changes in therapeutic intervention (with rationale)	not applicable
Follow-up and outcomes	10a	Clinician and patient-assessed outcomes (if available).	yes
	10b	Important follow-up diagnostic and other test results	not applicable
	10c	Intervention adherence and tolerability (How was this assessed?)	yes, via patient self-report
	10d	Adverse and unanticipated events	yes
Discussion	11a	A scientific discussion of the strengths AND limitations associated with this case report	yes
	11b	Discussion of the relevant medical literature with references	yes
	11c	The scientific rationale for any conclusions (including assessment of possible causes)	yes
	11d	The primary “take-away” lessons of this case report (without references) in a one paragraph conclusion	yes
Patient perspective	12	The patient should share their perspective in one to two paragraphs on the treatment(s) they received	yes, integrated into “Outcomes” section
Informed consent	13	Did the patient give informed consent? Please provide if requested	yes

### Appendix A.1. Transcript of Audio-Guided Recording

[Patient name omitted], I’m [instructor name omitted], I’m a good friend with [name omitted]. I’ve worked in a neurology clinic and researched breath techniques for migraines extensively.

This download will hopefully give you some relief and some skills to help you when you’re anticipating a migraine or enduring one. It’s a great practice to do when you’re not experiencing any migraines as well, just to keep your nervous system balanced.

The practice is really simple. All you need to do is be in a position of comfort. You can lay down in a dark room, you can have a weighted blanket, pillows under your knees. You can even lay on your side, or be sitting in a chair, however you feel most at ease.

We’re going to be lengthening the exhales gently. This starts to deactivate the stress response that contributes to the duration, intensity, and frequency of migraine experiences. It works chemically with the body in a similar way to certain medications, so this stuff is really really potent on the system, and you can trust it if you just allow your body to give you cues about what’s working and what’s not for you. If anything feels uncomfortable or strenuous, let it go.

And we’ll begin by just noticing your breath. Notice the position [of your body] and how the body is supporting the breath.

And if you’re not [already] using your nose gently, shift to breathing in and out with your nose. Let the jaw be relaxed, soften the bridge of the nose and soften the soft palate in the top of the mouth.

The air is flowing through nasal passageways that are right above the soft palate of the mouth. The roof of the mouth is the floor of the nose. As the air moves along the floor of the nose, it soothes the midbrain. Oxygen helps release tension by constricting vessels in the brain.

Exhaling for longer than you inhale deactivates the stress and tension response and helps create more parasympathetic healing tone across all of the vital systems in the body.

[Sound of ocean waves starts]

Just listen to the ocean sounds and allow your own breath to have an ocean-like sound, almost like you’re fogging a mirror through the nose. Don’t worry if you can’t get it exactly right, just practice controlling the volume of air without any tension to make a wave-like ocean sound, almost like a whisper, that further helps manage the chemistry in the brain and the body and tells the brain that all is in your control right now and everything is going to be okay.

Just trust the breath pattern we’re going to practice together, and listen to your own body, letting go any time you need to, returning when you feel ready.

Empty your lungs. When you’re ready to inhale, count 4 beats at a pace that feels easy for you and then exhale for six beats. Continue and just observe an internal knowing of how fast or slow to count.

At the end of each exhale, observe the pause at the bottom of the breath and see if you can allow the next inhale to happen for you whenever it’s ready.

In for 4, out for 6.

The breath is moving horizontally, which means the ribs are expanding outward on your inhales, and on the exhales the ribs come down and in along with the naval gently pulling towards the spine to finish the exhale all the way without force.

If you notice a long pause after the 6 count exhale, on your next exhale try counting for 8.

In for 4, out for 8.

On your inhales, the lower ribs lift and spread, and the collarbones lift and spread. The face, neck, and jaw are relaxed. On your exhales, you empty from the top down, the collarbones lower, the side ribs come in and down and the naval comes gently in and up. Continue on this pattern.

If it feels good, let’s add a pause at the top of the breath of 2 counts. In for 4 at your own pace, pause for 2, out for 6 or 8 counts—whatever’s comfortable.

And now I’ll let you continue in this pattern—in for 4, hold for 2, out for 6 or 8—with some ocean sounds in the background to help you relax and trust the wave of your breath.

Picture your tension and stress flowing out of the body on these exhales. At the bottom of the exhale is an empty void that allows the breath, body, and brain to reset itself. The inhales arise from this space without any force: let your inhales be relaxing, allowing the breath to spread and open in ways that feel easy and light. The pause at the top gives you a moment to soak in extra oxygen before releasing any tension or stress on the exhale. Inhaling ease, pausing to soak, releasing, letting go, starting over at the void.

As you continue practicing, you may be able to slow your count and continue to allow each inhale to emerge from the natural pause at the end of the exhale. Each breath is unique and the pause is sometimes really long and sometimes medium or short. Let it be organic.

[After 10 min of practice] We’ve come to the end of our formal practice. It’s a very simple tool you can take with you anywhere, any time to tune in and create the conditions from the inside out for your body to heal and reset, giving you relief from migraines and a chance to feel like yourself again.

### Appendix A.2. Written Instructions Sent to Patient via Email

As you’re learning this technique, choose a quiet place to practice, turn off all notifications and manage all interruptions. You can practice seated or lying down; whatever feels most comfortable. I recommend using headphones. It’s important to use the guidance as a tool at first to learn the technique while ultimately listening to your own body’s response. If at any time you feel like focusing on breathing is causing a sense of tension or air hunger, adjust the pace, soften the focus on breath, or let it go completely. You will benefit most by giving yourself time to rest and be with whatever arises. You can use this technique at the earliest signs of a migraine onset, as a daily practice to downregulate and support stability, or during a migraine headache. With practice you’ll learn how to use this technique without the download anytime you need to reset, downregulate, and manage stress-related symptoms. I look forward to hearing how it goes and please reach out if you have any questions!
